# *Surfactant protein B *polymorphisms are associated with severe respiratory syncytial virus infection, but not with asthma

**DOI:** 10.1186/1471-2466-7-6

**Published:** 2007-05-11

**Authors:** Beena Puthothu, Johannes Forster, Jessica Heinze, Andrea Heinzmann, Marcus Krueger

**Affiliations:** 1 University Children's Hospital, University of Freiburg, Mathildenstrasse 1, D-79106 Freiburg, Germany; 2 St. Josefs Hospital, Sautier Str. 1, D-79104 Freiburg, Germany

## Abstract

**Background:**

Surfactant proteins (SP) are important for the innate host defence and essential for a physiological lung function. Several linkage and association studies have investigated the genes coding for different surfactant proteins in the context of pulmonary diseases such as chronic obstructive pulmonary disease or respiratory distress syndrome of preterm infants. In this study we tested whether *SP-B *was in association with two further pulmonary diseases in children, i. e. severe infections caused by respiratory syncytial virus and bronchial asthma.

**Methods:**

We chose to study five polymorphisms in *SP-B*: rs2077079 in the promoter region; rs1130866 leading to the amino acid exchange T131I; rs2040349 in intron 8; rs3024801 leading to L176F and rs3024809 resulting in R272H. Statistical analyses made use of the Armitage's trend test for single polymorphisms and FAMHAP and FASTEHPLUS for haplotype analyses.

**Results:**

The polymorphisms rs3024801 and rs3024809 were not present in our study populations. The three other polymorphisms were common and in tight linkage disequilibrium with each other. They did not show association with bronchial asthma or severe RSV infection in the analyses of single polymorphisms. However, haplotypes analyses revealed association of *SP-B *with severe RSV infection (p = 0.034).

**Conclusion:**

Thus our results indicate a possible involvement of *SP-B *in the genetic predisposition to severe RSV infections in the German population. In order to determine which of the three polymorphisms constituting the haplotypes is responsible for the association, further case control studies on large populations are necessary. Furthermore, functional analysis need to be conducted.

## Background

Pulmonary surfactant consists of a lipid and protein complex, that is essential for normal lung function. The lipid complex lowers the surface tension at the air-liquid interface of the alveolus, and hereby prevents the lung from collapsing at low lung volume [1]. The protein complex participates in host defence mechanisms such as regulation of proinflammatory cytokine production, chemotaxis, or tissue repair [2]. So far five surfactant proteins (SP) have been identified in humans: SP-A1, SP-A2, SP-B, SP-C and SP-D. SP-A and SP-D are hydrophilic proteins and mainly involved in the innate immune system, whereas SP-B and SP-C are hydrophobic and essential constituents of lung surfactant. All surfactant proteins are mainly synthesised in the endoplasmic reticulum of alveolar type II cells. Because of its diverse functions, derangement in composition, structure, or function of surfactant proteins may lead to the development of a wide variety of pulmonary disorders.

Polymorphisms, especially in *SP-A *and *SP-B*, have been associated with lung diseases such as respiratory distress syndrome in preterm infants (RDS), congenital alveolar proteinosis (CAP) and chronic obstructive pulmonary disease (COPD) [3-5]. Furthermore, studies using knockout mice and clinical studies of human congenital proteinosis indicate that SP-B defiency is lethal [5].

*SP-B *is located on the short arm of human chromosome 2, it consists of 11 exons, whereby the 11^th ^exon is not translated [6]. There are two steps involved in the translation of SP-B: First, the *SP-B *transcription product, a 2000 bp long mRNA, is translated into the SP-B preprotein, which consists of 381 amino acids. Second, two proteolytic reactions take place that remove both the amino terminal and carboxy terminal arm of the proprotein and lead to the formation of the mature SP-B protein with a molecular mass of 8 kD [7].

RSV, a single-stranded RNA virus, is involved in at least 70% of cases of infectious infantile bronchiolitis and has been repetitively linked to asthma. It has been hypothesised that severe RSV infection in infancy might lead to the development of recurrent wheezing and/or bronchial asthma, and consequently a common genetic background of both diseases has been discussed [8-10].

In the present study, we aimed to verify the previously described polymorphisms in *SP-B *in the German population. Furthermore we were interested in whether any of these polymorphisms or haplotypes showed association with severe RSV associated diseases and/or pediatric asthma. We chose to study five single nucleotide polymorphisms (SNP) in *SP-B*: rs2077079 in the promoter region (-32G/T); rs1130866 resulting in the amino acid exchange T131I; rs2040349 in intron 8 (5781A/C); rs3024801 resulting in L176F and rs3024809 resulting in R272H.

## Methods

### Subjects

#### Population of infants with severe RSV infection

The population was recruited at the Centre for Pediatrics and Adolescent Medicine, Freiburg, Germany and at the Community Children's Hospital of Freiburg, St. Josefs Hospital. Infants were eligible when hospitalized due to RSV infection between September 1998 and March 2005. Infection with RSV was confirmed by antigen test and/or RSV-specific PCR [11]. According to the case definition children had symptoms of bronchiolitis, such as wheezing and tachypnea and needed either supplementary oxygen and/or gavage feeding and/or intravenous fluids. Children with congenital heart defects, immune deficiency, or chromosomal aberrations were excluded. DNA samples were collected either by blood taking or buccal smears with sterile cotton sticks. In total 131 children were included.

#### Asthmatic population

322 children with asthma (aged 5 to 18 years) were recruited from the South-Western part of Germany between July 2000 and January 2005. The patients were also characterised at the Centre for Pediatrics and Adolescent Medicine, Freiburg, Germany, using a standardized clinical protocol. An extended medical history was recorded including occurrence and duration of wheezing symptoms; previous and acute medications; severity of previous asthma attacks; previous allergic rhinitis or conjunctivitis; atopic dermatitis and any family history of allergic diseases.

The diagnosis was based on a clear-cut history of asthmatic symptoms, the use of anti-asthmatic medication and the presence of bronchial hyperreactivity. Bronchial hyperreactivity was defined as a fall in forced respiratory volume in one second (FEV1) by at least 15% in histamine testing or exercise provocation using standard protocols [12]. The exact recruitment procedure has been described in detail previously [13].

#### Control population

Two hundred and seventy randomly chosen probands were used as controls (aged 19 to 40 years). They originate from the same area in the South-Western part of Germany. No medical history was taken and no medical testing was performed on controls.

### Genotyping

DNA was extracted from peripheral blood leucocytes, or buccal smears following standard protocols and column purified (DNA midikit, Qiagen, Germany). Genotyping was performed by restriction fragment length polymorphism (RFLP). The polymorphisms under investigation and the conditions for the RFLP analyses (primer pairs, PCR conditions and restriction enzymes) are given in table 1.

**Table 1:** Primers, PCR conditions and restriction enzymes used for genotyping

**Polymorphism**	**Primer**	**PCR condition**	**Restriction enzyme**
**rs 2077079**	AGCCACAAGTCCAGGAACAT ATGCCTAGCACAAAGCAGTG	65°-55°C in -0.5°C steps, 20 cycles, 55°C 24 cycles	3U BSI1
**rs 1130866**	TGGGGGATTAGGGGTCAGTC CCATGGGTGGGCACAGGGG	65°-55°C in -0.5°C steps, 20 cycles, 55°C 20 cycles	1U TaaI
**rs2040349**	GACACTGGAGACGGAGGACT AAAGCCAGCTGATGCTCTTC	65°-55°C in -0.5°C steps, 20 cycles, 55°C 20 cycles	1U NIaIV
**rs 3024801**	CCTAACACTCCCACCCTGTG TCCTCCCCTCTCTCTTCCTC	65°-55°C in -0.5°C steps, 20 cycles, 55°C 20 cycles	pool sequencing
**rs 3024809**	GCTACTCCGTCATCCTGCTC GCCCATACCTTTTCCCTGTC	65°-55°C in -0.5°C steps, 20 cycles, 55°C 20 cycles	pool sequencing

### Sequencing

For each polymorphism three controls (homozygous wildtype, heterozygous and homozygous mutation) were sequenced by the dideoxy chain termination method [14] using the Big Dye Terminator cycle sequencing kit on an ABI 310 sequencer (Applied Biosystems). All the following studies included these reference individuals. In addition, pool sequencing was performed in order to detect the presence and allelic frequency of the polymorphism rs3024809 and rs3024801. The first pool comprises DNA from 24 asthmatic children and the second pool DNA from 27 asthmatic children. Thus, a total of 51 asthmatic children were included.

### Statistical analysis

For each SNP association analysis, based on the case-control design, was performed by using the Armitage's trend test. This test takes into account the individuals' genotypes rather than just the alleles, following the guidelines given by Sasieni [15], as implemented in the program Finetti (Thomas F. Wienker, unpublished data). The program Finetti was also used to calculate the Hardy Weinberg equilibrium (HWE) for each polymorphism in all populations. In addition, we performed haplotype frequency estimations with the programs FASTEHPLUS [16] and FAMHAP [17]. The extent of linkage disequilibrium between the polymorphisms has been calculated using the Arlequin program.

### Approval

The collection of blood and the experimental procedures were approved by the Ethical Committee of the University of Freiburg. A statement of informed consent was signed by all participants, or in the case of children, signed by their parents.

## Results

### Genotyping

Three polymorphisms – rs 2077079, rs1130866 and rs2040349 – were genotyped on 131 children with severe RSV infection, 270 controls and 322 asthmatic children. All polymorphisms were in Hardy Weinberg equilibrium in all populations (data not shown). Thus a systematic genotyping error or population stratification seems to be unlikely. In order to detect the occurrence and allelic frequency of the previously described polymorphisms rs3024809 and rs3024801 in our populations we performed pool sequencing. However, for both SNPs only the wild type was detectable in both pools. Thus, the polymorphisms seem to be very rare in our German population and were not investigated further.

### Association studies

The positions of the SNPs and the p-values for association with severe RSV infection and bronchial asthma obtained by Armitage's trend test are listed in table 2. None of the evaluated polymorphisms showed association with either disease.

**Table 2:** Polymorphisms under investigation

**Polymorphism**	**Position**	**RSV**	**Asthma**	**controls**	**RSV- controls**	**asthma- controls**	**asthma- RSV**
rs2077079	-32G/T	48; 56; 22	117; 139; 43	90; 137; 36	0.989	0.461	0.578
rs1130866	T131I	25; 70; 33	78; 133; 97	64; 126; 79	0.930	0.924	0.992
rs2040349	5781A/C	63; 69; 13	134; 139; 36	110; 116; 33	0.491	0.727	0.677

Genotype distribution in the populations (in the following order: homozygous wildtype, heterozygous, homozygous mutation). The p-value for association is listed as given by Armitage's trend test.

### Haplotype analyses and linkage disequilibrium

All polymorphisms were in tight linkage disequilibrium to each other in all populations (data not shown). The haplotypes occurring in the populations are listed in table 3. By typing three polymorphisms 2^3 ^haplotypes are theoretically possible; all of these exist in our populations. No significant differences in haplotype distribution were apparent between controls and the asthmatic population. In contrast, the haplotype distribution differed between the RSV population and controls. The most distinct difference was found for the haplotype bearing the wild type allele for all three polymorphisms (frequency of 0,23 in controls compared to 0,14 in the RSV population). The haplotype bearing two wildtype alleles and a mutated allele also showed a marked difference between the populations (see Table 3).

**Table 3:** Haplotypes in the three populations under investigation

**Haplotype**	**RSV**	**Controls**	**Asthma**
111	0,15	0,23	0,21
112	0,13	0,06	0,10
121	0,23	0,17	0,22
122	0,08	0,13	0,09
211	0,17	0,13	0,11
212	0,00	0,05	0,04
221	0,13	0,11	0,12
222	0,12	0,11	0,10

1 = wildtype, 2 = mutation. The polymorphisms are listed in the following order: rs2040349, rs1130866 and rs2077079.

Table 4 shows the results of haplotype analyses as calculated by FAMHAP and FASTEHP. Haplotypes were associated with severe RSV infection by p = 0,034 (FAMHAP) and p = 0,035 (FASTEHPLUS). In contrast, no association with bronchial asthma was detected.

**Table 4:** Results of the haplotype analyses using the programs FAMHAP and FASTEHP

	**RSV-controls**	**Asthma-Controls**	**RSV-asthma**
FAMHAP	0,034	0,396	0,12
FASTEHP	0,035	0,364	0,09

## Discussion

Surfactant proteins play important roles in the maintenance of normal lung function and host defence. Acute or chronic inflammation may lead to local alteration of SP concentrations. Our study focussed on the genetic variability of these proteins in an attempt to identify children at increased risk for inflammatory lung disease like severe RSV infection and bronchial asthma. Thus the aim was to determine, whether polymorphisms within *SP-B *show association with one or both diseases. Furthermore, we were interested to detect a common genetic link between both diseases – as has previously been discussed [8-10].

However, we did not find any association of three common *SP-B *polymorphisms neither with severe RSV infections nor with bronchial asthma. In addition, haplotype analysis of the asthmatic population supported further that *SP-B *polymorphisms do not play a major role in the development of asthma. This is an important point according to the biophysical and immunological properties of surfactant proteins in lung development and function [18]. Hohlfeld and colleagues demonstrated alteration of all SPs (SP-A, SP-B, SP-C and SP-D) in the bronchoalveolar lavage after segmental allergen challenge in patients with asthma. In addition, they assume that activation of the surfactant system is not a universal response to airway inflammation, but rather a specific response to the disturbance of surfactant function by allergen challenges [19].

Haplotype analysis of the three *SP-B *polymorphisms in the RSV population suggests an influence of these SNPs on the genetic predisposition to severe RSV infection (p = 0.034 and p = 0.035, respectively). Interestingly, we have already described association of Surfactant protein C with RSV bronchitis in the same cohort [20]. This might further emphasis the importance of the Surfactant system in RSV bronchiolitis. However, looking at the haplotype distribution, it is not possible to decide which haplotype may be the most important one in the development of severe RSV infections. Here functional studies are clearly needed.

This is the first analysis of *SP-B *polymorphisms in RSV associated diseases. Previous studies have implicated *SP-B *polymorphisms as contributing factors to RDS [21], COPD and acute respiratory failure [22]. In the majority of published studies the amino acid variant T131I is associated with increased risk to pulmonary diseases. Interestingly, the amino acid change T131I leads to the block of the N-glycosylation site [23]. This change may have an impact on SP-processing and/or on the function of SP-B. Nevertheless, we did not find association of T131I with severe RSV infection. This could be due to the small sample size of our RSV population. Still our population is larger than the ones used in the above mentioned studies. In addition, we performed power calculations to test the validity of our results. In our asthmatic population with 322 individuals the power to find association with asthma (p-value of p = 0.05, assuming a relative risk of the variant of 2) is between 0.94 for rs2077079 and 0.97 for rs2040349. Regarding the RSV population the power is slightly lower due to the smaller sample size (0.83 to 0.88).

The polymorphisms rs2077079 did not show association with asthma or severe RSV infection in our populations. One previous study evaluated this polymorphism in a population with RDS and reported association of this polymorphism with RDS in black subjects [21]. However, the population size was too small to yield significant results. No significant association was observed between the SNP and RDS in the white population. This is in line with our results. As so little is known about the functional impact of this SNP, we can only speculate about its role in haplotype distribution.

## Conclusion

In conclusion, our results indicate a possible association of *SP-B *haplotypes with severe RSV infection in the German population. In order to determine which one or ones of the three polymorphisms forming the haplotypes is/are responsible for the association, further case control studies on large populations are necessary. Furthermore, functional analysis needs to be conducted.

## Competing interests

The author(s) declare that they have no competing interests.

## Authors' contributions

BP performed genotyping and sequencing of *SBP *polymorphisms as well as the statistical analyses and drafted the manuscript.

JF participated in the clinical design of the study.

JH performed DNA extraction of the study populations.

AH conceived and coordinated the study and helped to draft the manuscript.

MK participated in the clinical design of the study and the recruitment of the RSV population.

All authors read and approved the final manuscript.

## Pre-publication history

The pre-publication history for this paper can be accessed here:



## References

[B1] Johansson J, Curstedt T (1997). Molecular structures and interactions of pulmonary surfactant components. Eur J Biochem.

[B2] Phelps DS (2001). Surfactant regulation of host defense function in the lung: a question of balance. Pediatr Pathol Mol Med.

[B3] Nogee LM, Garnier G, Dietz HC, Singer L, Murphy AM, deMello DE, Colten HR (1994). A mutation in the surfactant protein B gene responsible for fatal neonatal respiratory disease in multiple kindreds. J Clin Invest.

[B4] Lewis JF, Jobe AH (1993). Surfactant and the adult respiratory distress syndrome. Am Rev Respir Dis.

[B5] Lin Z, deMello DE, Wallot M, Floros J (1998). An SP-B gene mutation responsible for SP-B deficiency in fatal congenital alveolar proteinosis: evidence for a mutation hotspot in exon 4. Mol Genet Metab.

[B6] Vamvakopoulos NC, Modi WS, Floros J (1995). Mapping the human pulmonary surfactant-associated protein B gene (SFTP3) to chromosome 2p12--> p11.2. Cytogenet Cell Genet.

[B7] Hawgood S, Derrick M, Poulain F (1998). Structure and properties of surfactant protein B. Biochim Biophys Acta.

[B8] Sigurs N, Gustafsson PM, Bjarnason R, Lundberg F, Schmidt S, Sigurbergsson F, Kjellman B (2005). Severe respiratory syncytial virus bronchiolitis in infancy and asthma and allergy at age 13. J Respir Crit Care Med.

[B9] Sigurs N, Bjarnason R, Sigurbergsson F, Kjellman B (2000). Respiratory syncytial virus bronchiolitis in infancy is an important risk factor for asthma and allergy at age 7. Am J Respir Crit Care Med.

[B10] Stein RT, Sherrill D, Morgan W, Holberg CJ, Halonen M, Taussig LM, Wright AL, Martinez FD (1999). Respiratory syncytial virus in early life and risk of wheeze and allergy by age 13 years. Lancet.

[B11] Forster J, Ihorst G, Rieger CH, Stephan V, Frank HD, Gurth H (2004). Prospective population-based study of viral lower respiratory tract infections in children under 3 years of age (the PRI.DE study). Eur J Pediatr.

[B12] (1998). Worldwide variation in prevalence of symptoms of asthma, allergic rhinoconjunctivitis, and atopic eczema: ISAAC. The International Study of Asthma and Allergies in Childhood (ISAAC) Steering Committee. Lancet.

[B13] Heinzmann A, Jerkic SP, Ganter K, Kurz T, Blattmann S, Schuchmann L, Gerhold K (2003). Association study of the variant Arg110Gln in Interleukin-13 with atopic diseases and juvenile idiopathic arthritis. J All Clin Immunol.

[B14] Sanger F, Nicklen S, Coulson AR (1992). DNA sequencing with chain-terminating inhibitors. Biotechnology.

[B15] Sasieni PD (1997). From genotypes to genes: doubling the sample size. Biometrics.

[B16] Zhao JH, Sham PC (2002). Faster haplotype frequency estimation using unrelated subjects. Hum Hered.

[B17] Becker T, Knapp M (2004). Maximum-likelihood estimation of haplotype frequencies in nuclear families. Genet Epidemiol.

[B18] Revak SD, Merritt TA, Degryse E, Stefani L, Courtney M, Hallman M, Cochrane CG (1988). Use of human surfactant low molecular weight apoproteins in the reconstitution of surfactant biologic activity. J Clin Invest.

[B19] Erpenbeck VJ, Schmidt R, Gunther A, Krug N, Hohlfeld JM (2006). Surfactant protein levels in bronchoalveolar lavage after segmental allergen challenge in patients with asthma. Allergy.

[B20] Puthothu B, Krueger M, Heinze J, Forster J, Heinzmann A (2006). Haplotpyes of Surfactant Protein C are associated with common paediatric lung diseases. Pediatr Allergy Immunol.

[B21] Floros J, Fan R, Diangelo S, Guo X, Wert J, Luo J (2001). Surfactant protein (SP) B associations and interactions with SP-A in white and black subjects with respiratory distress syndrome. Pediatr Int.

[B22] Seifart C, Plagens A, Brodje D, Muller B, von Wichert P, Floros J (2002). Surfactant protein B intron 4 variation in German patients with COPD and acute respiratory failure. Dis Markers.

[B23] Jacobs KA, Phelps DS, Steinbrink R, Fisch J, Kriz R, Mitsock L, Dougherty JP, Taeusch HW, Floros J (1987). Isolation of a cDNA clone encoding a high molecular weight precursor to a 6-kDa pulmonary surfactant-associated protein. J Biol Chem.

